# In vivo dynamic intrusion and extrusion of the menisci in varus and valgus load within a healthy population

**DOI:** 10.1002/jeo2.70325

**Published:** 2025-08-13

**Authors:** Sebastian F. Bendak, Joachim Georgii, Elham Taghizadeh, Stefan Heldmann, Hans Meine, Thomas Lange, Jonas Buchholtz, Andreas Fuchs, Moritz Mayr, Hagen Schmal, Kaywan Izadpanah

**Affiliations:** ^1^ Department of Orthopedic Surgery and Traumatology, Freiburg University Hospital Albert‐Ludwigs‐University Freiburg Freiburg Germany; ^2^ Fraunhofer Institute for Digital Medicine MEVIS Bremen Germany; ^3^ Department of Diagnostic and Interventional Radiology, Division of Medical Physics, Faculty of Medicine Albert‐Ludwigs‐University of Freiburg Freiburg Germany

**Keywords:** dynamic MRI evaluation, in vivo MRI, meniscal movement, varus and valgus load

## Abstract

**Purpose:**

Axial loading, varus and valgus stress lead to meniscal motion towards the joint periphery, defined as meniscal extrusion. Direction and amount of extrusion is unknown as this is a dynamic process within a 3D environment dependent on joint loading as well as individual anatomy. We propose that there is motion in all compartments of the medial and lateral meniscus during valgus and varus stress.

**Method:**

MRI scans of 31 healthy subjects in varus or valgus stress positions were acquired with the help of a pneumatic loading device. Semiautomatic segmentation of the menisci, the femur and the tibia (with corresponding cartilages) was carried out. An individual 3D model of the joint was generated. The meniscal movement was calculated within a tibia‐based coordinate system and broken down into total and partial meniscal movement (anterior/posterior horn, intermediate part).

**Results:**

Under valgus load the medial meniscus (MM) showed average movement of 1.5 (±0.5) mm in lateral‐posterior direction with most lateral motion of 1.4 (±0.7) mm in the intermediate part. The lateral meniscus averaged 1.6 (±1.0) mm in lateral‐anterior motion, exhibiting maximal lateral motion in the anterior horn (AH) 0.7 (±0.8) mm and posterior horn 0.6 (±0.6) mm. In response to the varus load, average MM motion was 0.9 (±0.5) mm in medial‐anterior direction with the largest medial movement in the AH 0.9 (±1.1) mm. The lateral meniscus moved in average 1.6 (±0.8) mm into lateral‐posterior direction with the intermediate part showing the largest medial motion of 0.6 (±0.4) mm.

**Conclusion:**

In a healthy population, the menisci extrude up to 1.5 mm during varus and valgus loading. The anterior and posterior horn show greater dynamic extrusion than the intermediate part. However, an in vivo dynamic intrusion mechanism of meniscus when discharged (medial 1.45 mm, lateral 1.56 mm) could be demonstrated. Quantification and reconstruction of this phenomenon might be of crucial importance during meniscal root or meniscal transplantation surgery.

**Level of Evidence:**

Level II, descriptive laboratory study.

**Abbreviations**
AH: anterior horn
AHLM: anterior horn lateral meniscus
AHMM: anterior horn medial meniscus
AP: anterior‐posterior
IP: intermediate part
IPLM: intermediate part lateral meniscus
IPMM: intermediate part medial meniscus
LM: lateral meniscus
ML: medio‐lateral
MM: medial meniscus
MPT: moiré phase tracking
PH: posterior horn
PHLM: posterior horn lateral meniscus
PHMM: posterior horn medial meniscus
PMC: prospective motion correction
SSD: sum‐of‐squared‐differences

## INTRODUCTION

Motion in the knee joint greatly affects the meniscal position. The effect of valgus or varus stress on the motion of the meniscus shows significant peripheral movement on the compressed side [33]. The state of the external meniscal ring outside the joint is called extrusion.

Extrusion is defined as the meniscal part exterior to the edge of tibiofemoral cartilage not interacting in the joint [1]. Its role in the development and progression of osteoarthritis has been extensively discussed [15, 17, 27, 34].

The degree of extrusion between loaded and unloaded state can be described as dynamic extrusion. The absence of this motion or displacement of the meniscus under load can reflect on the condition of the meniscus and its function [6, 11, 12, 18].

Extrusion as well as dynamic extrusion are altered by meniscal tears, degenerative changes and varus/valgus alignment. Strong association of cartilage damage, bone marrow oedema and osteoarthritis with an extruded meniscus can be identified [2, 7, 9, 29, 38].

Studies show that preserving a meniscus and reconstructing its anatomy with surgical repair techniques after trauma will reduce extrusion and decelerate progression of osteoarthritis [10, 20, 21, 24, 30, 31, 32, 44].

To gain new insights of biomechanical behaviour in vivo and deepen our comprehension on how this structure influences the knee, it is crucial to evaluate the meniscal dynamics in stress positions under load and when discharged.

Both understanding more about the in vivo motion of the meniscus and establishing a baseline for comparison will help to understand the effect of pathologies and to answer questions about limitations in rehabilitation, formation and progression of osteoarthritis as well as the needs and possible impact of surgical procedures.

In this study, the aim is to compute the in vivo movement of the meniscus in healthy adults with the help of an in vivo MRI‐based 3D knee joint model during application of valgus or varus stress.

We hypothesise that there is movement in all compartments of the compressed meniscus towards the outer edge of the tibial plateau and of the discharged meniscus towards the centre of the joint.

## MATERIALS AND METHODS

### Cohort selection

For this study a population of 31 healthy subjects (15 women and 16 men) was recruited. The mean age was 25.6 ± 4.4 years. Mean height and weight were 176.8 ± 9.1 cm/71.2 ± 10.9 kg resulting in a mean body mass index (BMI) of 22.7 ± 1.9. Thirteen right knees and 18 left knees were included in the present study.

The examined knee had to be free of the following: any previous surgery; chronic pain; past trauma; signs of any degenerative changes or lesions on bone, cartilage, meniscus or ligaments.

The acquired MR imaging also confirmed no visible injury.

### Data acquisition

For image acquisition, a Magnetom Trio 3T system (Siemens Healthineers) with an eight‐channel multipurpose coil (NORAS MRI products) was used.

All MRI scans were performed with a T1‐weighted spoiled 3D gradient‐echo sequence using slab‐selective water excitation and covering a field‐of‐view (FOV) of 145 mm (anterior‐posterior [AP]) × 125 mm (right/left [RL]) × 160 mm (foot/head [FH]) with a spatial resolution of 0.6 mm (AP) × 0.5 mm (RL) × 0.6 mm (FH). Further sequence parameters were TR = 16 ms, TE = 6.88 ms, exc. angle = 15°, readout bandwidth = 130 Hz/Px, readout direction = AP. The MRI scan duration was 5:34 min.

To mitigate motion artifacts and ensure acceptable image quality, the MRI sequence was augmented with prospective motion correction (PMC) based on a moiré phase tracking (MPT) system (Metria Innovation Inc.) [22, 23]. The camera setup was implemented in other studies investigating knee kinematics with MRI as well [35, 36].

**Figure 1:**
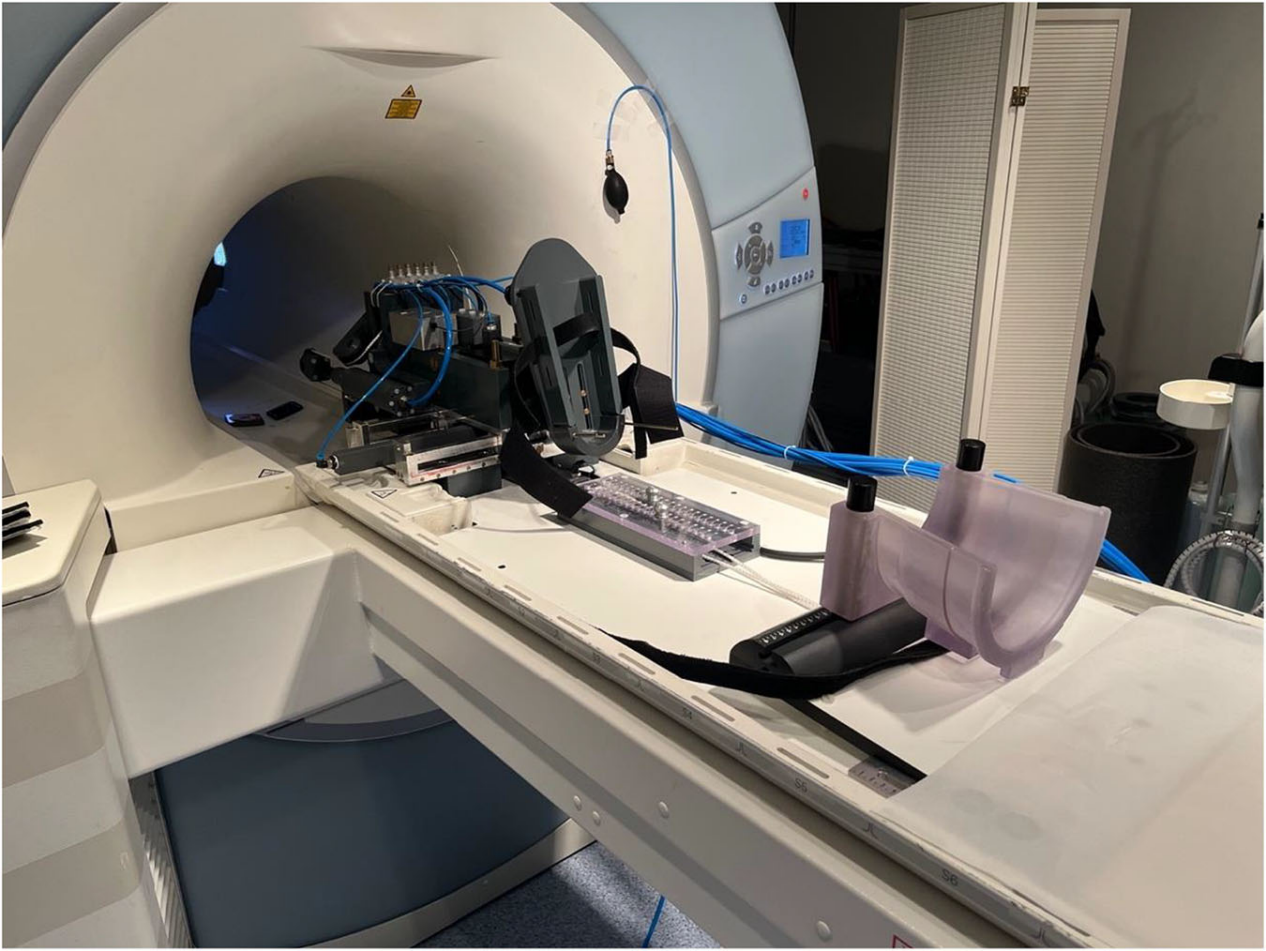
Pneumatic loading device. Femoral fixation could be elevated with a pneumatic pillow. The foot piece was able to retrieve with 5 N and lateralise with 50 N.

For the MRI scans, the examined leg was placed in a MR‐compatible pneumatic device, which enabled the knee to be brought into the stress positions of interest (see Figure 1). Individual adjustments were made depending on height and body proportions. Correct foot and femoral fixation were ensured.

The device was controlled and monitored from the console room. It achieved 20° knee flexion by elevating the thigh with the femoral fixation and retracting the foot piece with 5 N to maintain heel contact. With the subject in place, scans were performed with 50 N of valgus or varus stress induced by the corresponding lateralisation of the foot piece. The same setup was used to validate a finite element simulation for predicting individual knee joint kinematics [39] and to investigate rotational meniscal motion [14].

### 3D MRI postprocessing

**Figure 2:**
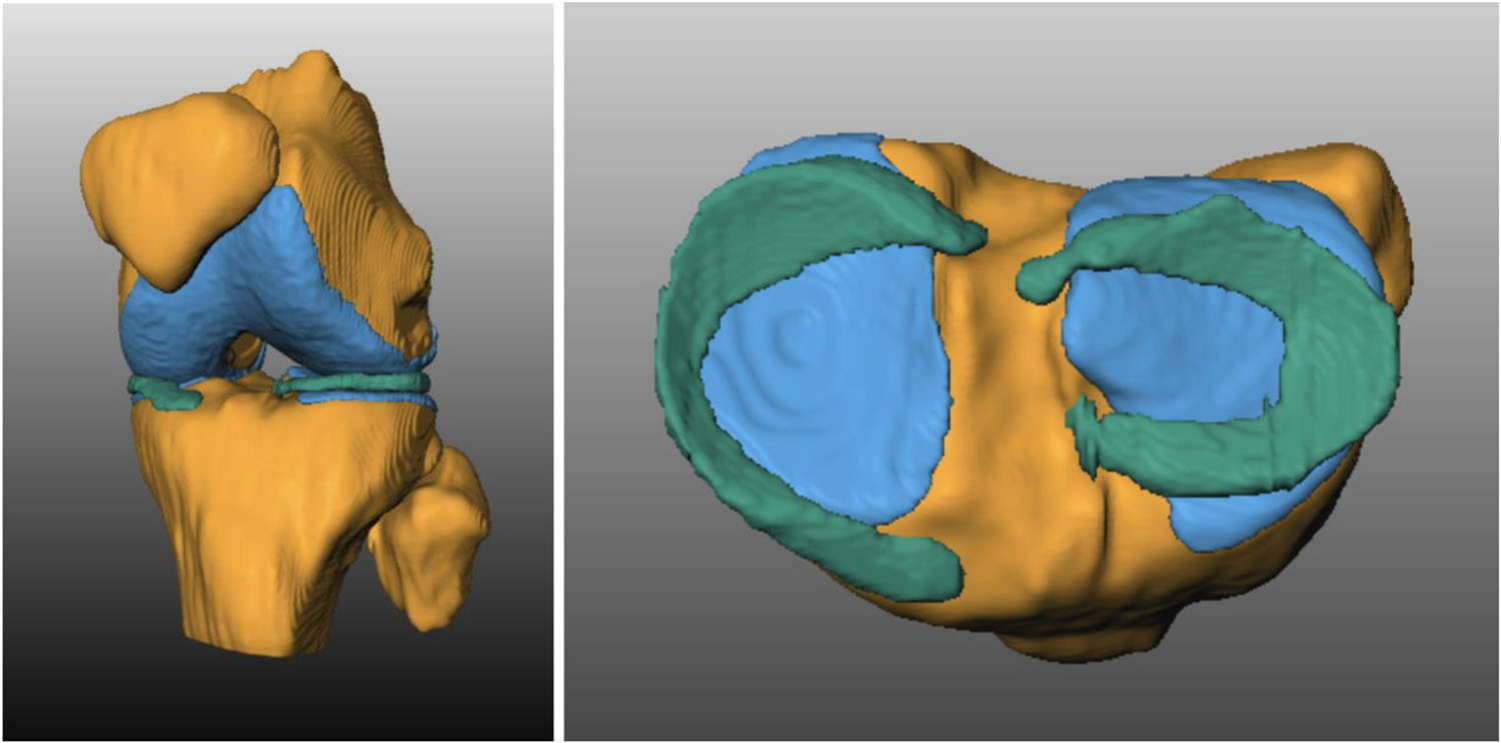
3D knee model. Semiautomated segmentation model exemplary participant.

For all MR images, semiautomatic annotations of the femoral and tibial bones and cartilages as well as of the lateral and medial menisci were used. Segmentation models were trained on these annotations, and manual corrections were utilised to iteratively improve the model accuracy. All structures were validated by clinical experts prior to the analysis (see Figure 2).

An image‐based registration pipeline was used to analyse the meniscal displacements between different positions. Rigid registration restricted to the tibial bone mask was applied to initially align the images. As a result, all MR images of a single subject were available in a consistent coordinate frame to analyse the motion relative to a fixed tibia.

Next, a nonlinear registration approach was used to compute the meniscal movement. It is based on a common variational approach utilising a curvature‐based regularisation of the computed deformation field [13]. To measure similarity, the sum‐of‐squared‐differences (SSD) was used on the image values in the meniscal regions. To enforce reasonable volume overlap, an additional penalty term was used. It measures the point‐to‐point distance between the surfaces, which is efficiently computed by a distance transformation of the menisci contours [3]. The approach showed good convergence even in cases where the initial volume overlap between the meniscal shapes is very low (which is naturally the case for large movements). A multilevel approach based on a matrix‐free Gauss–Newton method was used to numerically solve the respective optimisation problem. In this way, local minima could be effectively overcome in the solution process. As a result, the method provides a dense deformation field representing the motion each point (voxel) in the menisci masks has taken. Deformation vectors outside the meniscal regions have been cropped prior to the analysis of the motion.

**Figure 3:**
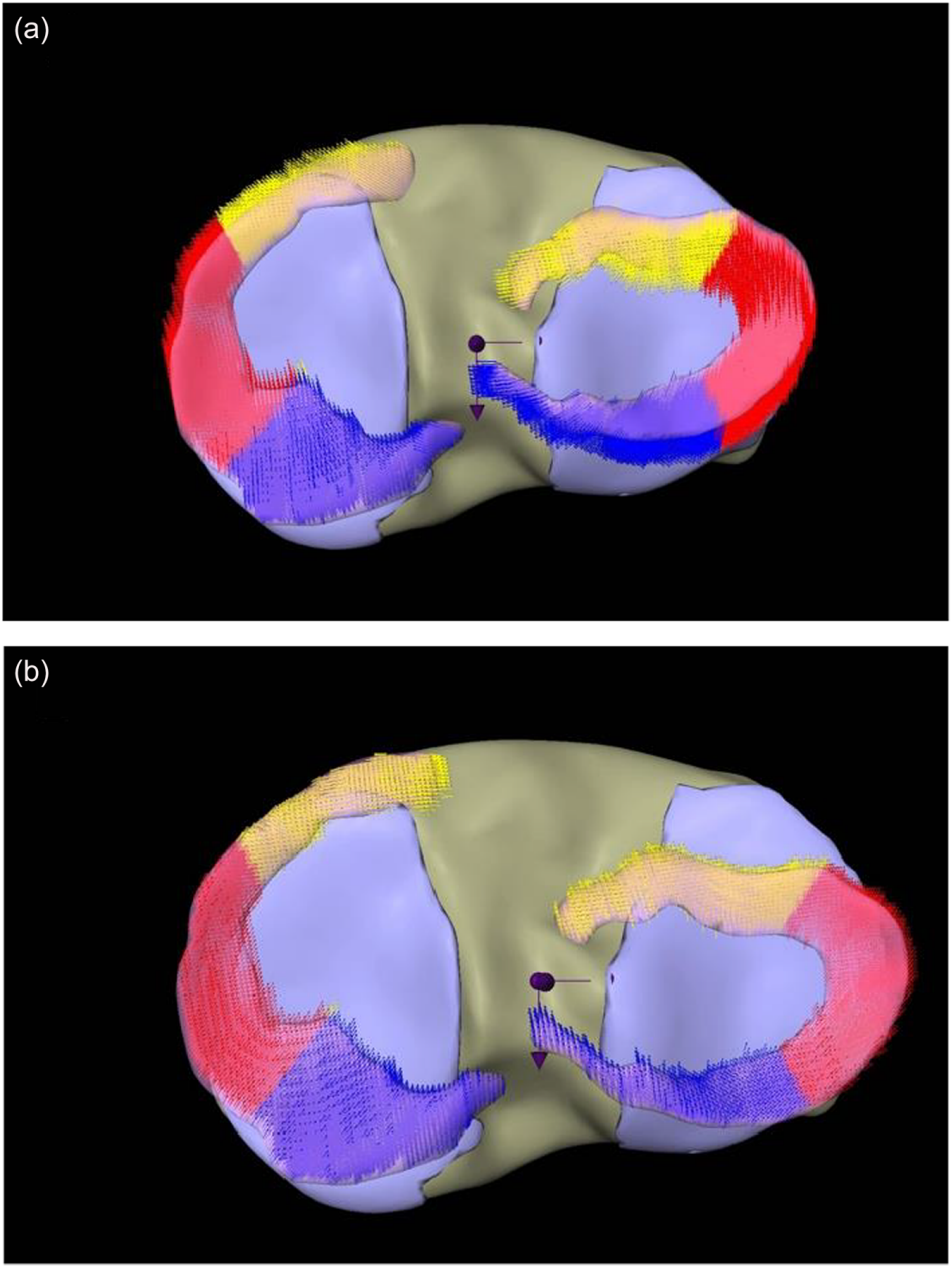
(a, b) Representation of all calculated motion vectors in both menisci. (a) Medial meniscal motion during varus load in an exemplary participant. (b) Lateral meniscal motion during valgus load in an exemplary participant. Caption: left: medial meniscus; right: lateral meniscus; yellow: anterior horn; red: intermediate horn; blue: posterior horn; central purple arrows represent the coordinate axis in mediolateral and anteroposterior direction.

For further analysis, the menisci were partitioned into three parts with equal length of the arch. For each of these partitions, the average motion was computed from all respective voxels. Additionally, these 3D motion vectors were projected onto the AP and the medio‐lateral axis. These axes are computed by a principal component analysis of all points of the medial and lateral menisci in unloaded position (see Figure 3a,b).

### Statistical analyses

In this study the descriptive statistics are given as mean values and standard deviations. With R Studios statistical analyses were performed and interpreted in an exploratory sense. There was no multiple testing carried out. Therefore, adjustment for multiple testing was not applicable in this exploratory study.

## RESULTS

**Figure 4:**
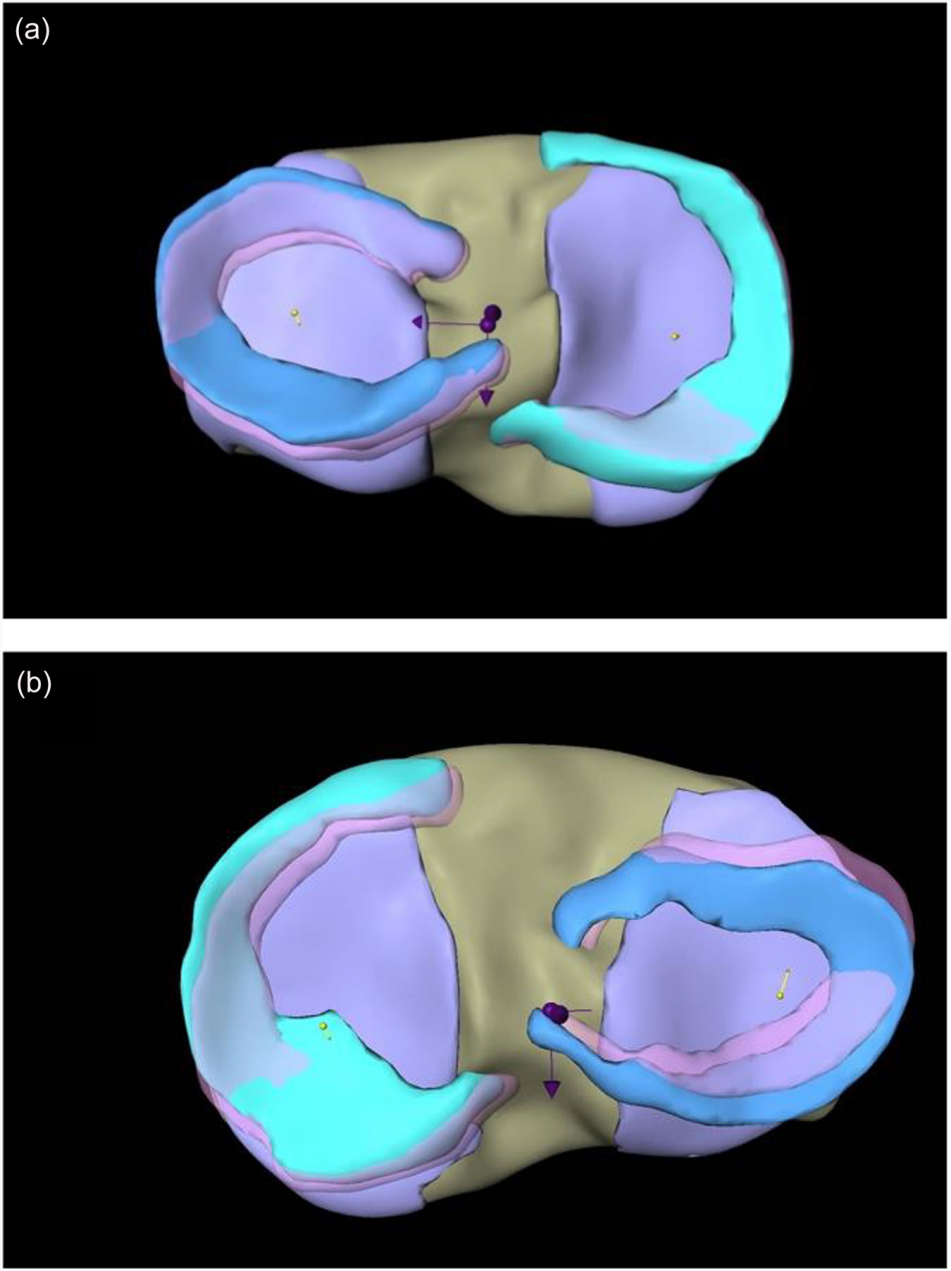
(a, b) Representing combined vectors of movement for complete medial meniscus (MM) and lateral meniscus (LM). (a) Medial meniscal motion of the MM and medio‐posterior meniscal motion of the LM during varus load in an exemplary participant. (b) Latero‐posterior meniscal motion of the MM and latero‐anterior meniscal motion of the LM during valgus load in an exemplary participant. Caption: green: MM; blue: LM; yellow arrows: overall meniscus motion of MM and LM; central purple arrows represent the coordinate axis in mediolateral and anteroposterior direction.

**Figure 5:**
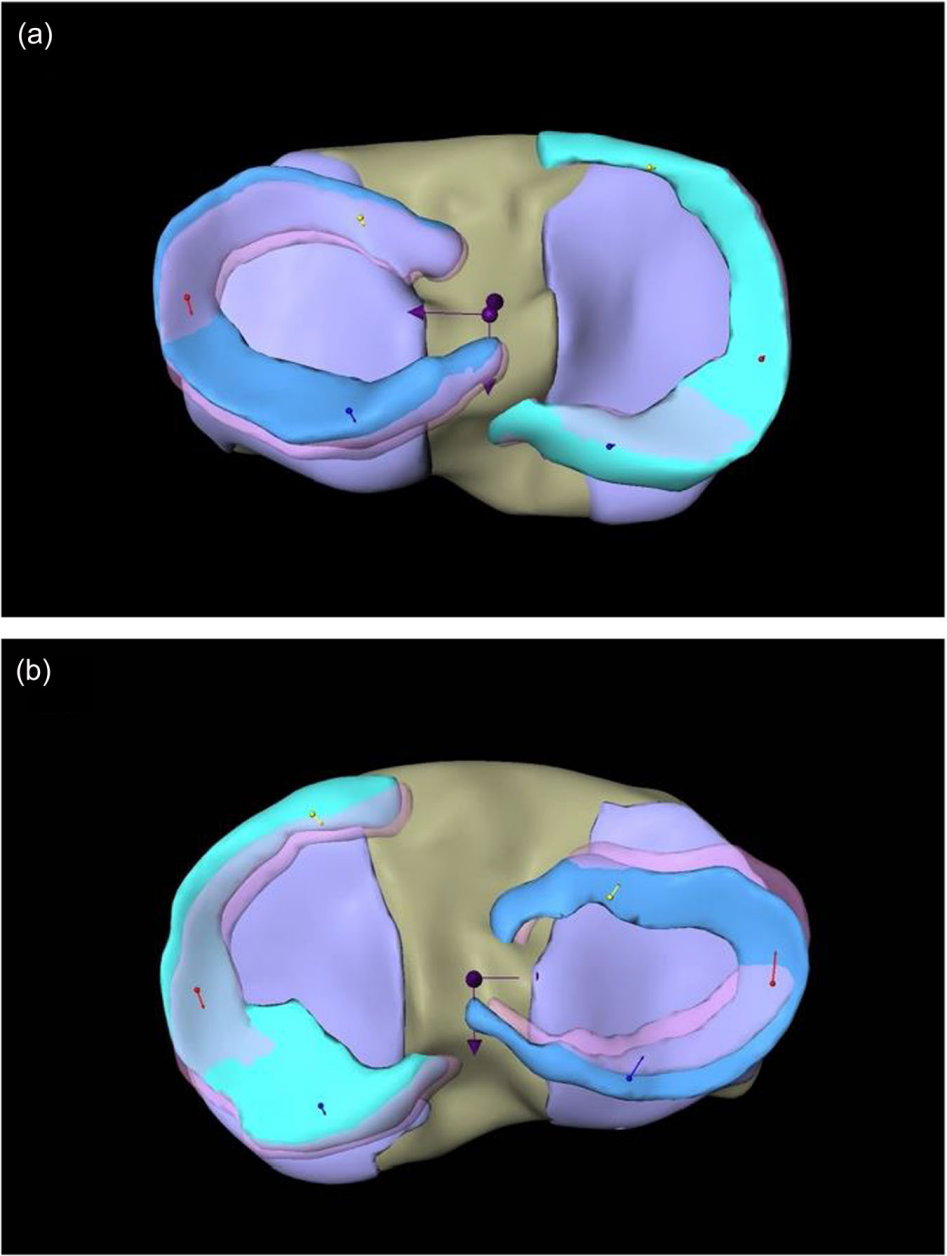
(a, b) Representing combined vectors in anterior, intermediate and posterior horn of lateral meniscus (LM) and medial meniscus (MM) motion. (a) Meniscal motion in varus load subdivided in anterior/posterior horn and intermediate part in an exemplary participant. (b) Meniscal motion in valgus load subdivided in anterior/posterior horn and intermediate part in an exemplary participant. Caption: green: MM; blue: LM; yellow arrows: meniscus movements in the AH of LM and MM; red arrows: meniscus movements in the IP of LM and MM; blue arrows: meniscus movements in the PH of LM and MM; central purple arrows represent the coordinate axis in mediolateral and anteroposterior direction.

The movements of the complete menisci (medial meniscus [MM]; lateral meniscus [LM]; see Figure 4a,b), as well as their separate segments (anterior horn [AH]; intermediate part [IP]; posterior horn [PH]; see Figure 5a,b) are computed and displayed as the total vectors length (Avg) together with movement in AP and medio‐lateral direction (ML). The length of the vector respectively represents the movement in millimetres.

### MM, varus load

The MM moved on Avg 0.85 (SD ± 0.53) mm, as the result of 0.47 (SD ± 0.48) mm in medial and 0.1 (SD ± 0.72) mm in anterior direction. Most movement in ML direction is detected in the AH with 0.86 (SD ± 1.05) mm. Analysis of the other meniscal subgroups can be found in Table 1.

**Table 1:** Total meniscus movements (in mm) varus and valgus load.

Total meniscus movements (in mm) varus/valgus						
	Varus			Valgus		
	Avg	ML	AP	Avg	ML	AP
Medial meniscus						
Overall	0.846 (±0.53)	−0.468 (±0.48)	−0.102 (±0.72)	1.453 (±0.53)	1.096 (±0.52)	0.625 (±0.64)
AH	1.189 (±0.92)	−0.860 (±1.05)	0.082 (±0.55)	1.868 (±0.70)	1.057 (±0.85)	1.142 (±0.64)
IP	0.941 (±0.63)	−0.374 (±0.48)	0.006 (±0.95)	1.913 (±0.71)	1.440 (±0.68)	0.868 (±0.86)
PH	0.805 (±0.57)	−0.333 (±0.44)	−0.291 (±0.75)	1.195 (±0.47)	0.879 (±0.57)	0.150 (±0.70)
Lateral meniscus						
Overall	1.563 (±0.81)	−0.300 (±0.45)	0.871 (±1.42)	1.560 (±1.01)	0.541 (±0.47)	−1.272 (±1.14)
AH	1.672 (±0.81)	−0.225 (±0.70)	1.143 (±1.18)	1.409 (±0.91)	0.674 (±0.78)	−0.777 (±1.03)
IP	1.945 (±1.02)	−0.548 (±0.41)	0.991 (±1.83)	1.727 (±1.31)	0.351 (±0.31)	−1.553 (±1.43)
PH	1.352 (±0.84)	−0.056 (±0.53)	0.444 (±1.34)	1.668 (±0.91)	0.643 (±0.57)	−1.340 (±0.94)

*Note*: A positive value in ML direction corresponds to a movement in lateral direction (negative in medial direction); a positive value in AP direction corresponds to a movement in posterior direction (negative in anterior direction). Values in parentheses represent standard deviations.

Abbreviations: AH, anterior horn; AP, meniscus movement in anteroposterior direction; Avg, average, total length of motion vector; IP, intermediate horn; ML, meniscus movement in mediolateral direction; PH, posterior horn, all values are given in mm.

### MM, valgus load

In valgus load the MM showed on Avg 1.45 (SD ± 0.53) mm movement, as the result of 1.01 (SD ± 0.52) mm in lateral and 0.63 (SD ± 0.64) mm in posterior direction. Greatest motion in ML direction here had the IP with 1.44 (SD ± 0.68) mm. Analysis of the other meniscal subgroups can be found in Table 1.

### LM, varus load

During varus load, the LM displayed Avg motion of 1.56 (SD ± 0.81) mm, as the result of a medial component of 0.3 (SD ± 0.45) mm and posterior of 0.87 (SD ± 1.42) mm. The IP moved most in ML direction with 0.55 (SD ± 0.41) mm. Analysis of the other meniscal subgroups can be found in Table 1.

### LM, valgus load

In valgus load, the LM Avg was 1.56 (SD ± 1.01) mm, as the result of 0.54 (SD ± 0.47) mm in lateral and 1.27 (SD ± 1.14) mm in anterior direction. Both AH with 0.67 (SD ± 0.78) mm and PH with 0.64 (SD ± 0.57) showed great movement in ML direction. Analysis of the other meniscal subgroups can be found in Table 1.

## DISCUSSION

This study shows not only peripheral movement on the compressed loaded side but also in the unloaded discharged compartment, specifically, retractive motion to the joint's centre, hereinafter called dynamic intrusion. Interestingly, the MM showed further retraction when unloaded and greater motion between loaded (0.47 mm) and discharged (1.01 mm) condition in ML direction, where the LM displayed more Avg motion with 1.56 mm discharged and 1.56 mm loaded. This dynamic intrusion, when discharged shows the elastic recoil force of the meniscus. This force allows the meniscus to stay in contact with the femur and tibia for a longer period and distance. The prolonged contact might cushion administered forces better, preventing peak contact pressure that could otherwise cause damage to the joint, specifically to the cartilage or the meniscus itself. Loss of dynamic intrusion by trauma or degeneration could contribute to accelerated deterioration of the joint.

When loaded with varus stress the MM had a dynamic overall extrusion of 0.47 mm in ML direction with the greatest motion of 0.86 mm in the AH. The varus loads shifted AH 0.08 mm and PH 0.30 mm towards each other. This movement resulted in a further change of the meniscal shape: as the AH and PHs move closer together, the C‐shape of the meniscus straightens, and the curvature increases. The motion of the MM thereby was not entirely achieved by elongation of collagenous fibres but also by plasticity of the meniscal structure.

The LM in varus stress showed great average motion of 1.56 mm even when discharged. The LM retracted discharged into a posteromedial direction, as a dynamic intrusion, particularly in the AH (1.67 mm) and the IP (1.95 mm).

When discharged in valgus position, the MM was drawn toward the centre of the joint in all segments, with a high average dynamic intrusion of 1.87 mm in the AH and 1.91 mm in the IP. The main ML movement of the dynamic intrusion here was in the IP with 1.44 mm.

Applied valgus load caused the LM to extrude in ML direction with the main motion residing in the AH (0.67 mm) and the PH (0.64 mm).

Overall, both menisci adapted to load into a ML direction mainly with motion in the AH and PH. During discharge of the compartment the greatest effect on ML motion was in the intermediate horn in this cohort.

Differences in meniscal position and motion are especially seen in patients with osteoarthritis. In a 2‐year unloaded supine MRI follow up of arthritis patients, Bloecker et al. detected significant changes in extrusion with a 3D‐based analysis [4]. In loaded condition Stehling et al. discovered that the meniscus adapts differently in degenerative joints compared to healthy ones by extruding further to the outside [37].

Radial meniscal tears such as posterior meniscal root tears also have a large influence on the meniscal extrusion. When tears disrupt the meniscus circular fibres, the meniscus loses its function in relation to the extent of the defect. The result is a decreased contact area with increased contact pressure [7, 28, 29].

Determining the dynamic extrusion can also be useful diagnostically in meniscal root tears or other‐configurated meniscal tears, hence showing clinical purpose. The menisci's unloaded position and their loaded motion combined seem to be relevant here [6, 11, 12, 18, 32].

After meniscal root repair or meniscal transplantation extrusion can still be an issue and impede with a good outcome by posing the risk of developing osteoarthritis [19, 25]. Surgical techniques have been developed to decrease the amount of extrusion [10, 20, 21, 24]. For example, when the meniscotibial ligament is ruptured, reconstruction was able to reduce the by injury developed extrusion [26, 31].

Studies investigating meniscal motion in vivo and with load have used open MRI because the limited space of a closed bore could not provide for adequate controlled movement [5, 43].

By inducing motion in a controlled manner, the MRI‐compatible device used in this study permitted imaging of loaded positions while staying inside the bore of a 3T MRI.

Motion artifacts caused by putting a joint under stress were reduced and good image quality was ensured with prospective motion correction [22]. Such image quality was essential for evaluation and semiautomated segmentation.

In most previous studies, MR imaging measurements were performed with a single‐slice method to determine the meniscal position [6, 9, 44]. Especially, longitudinal cohorts benefited from this time‐efficient method [38]. Depending on execution and slice selection, this method could under‐ or overestimate meniscal extrusion [16]. Evaluating only one slice and one edge of a three‐dimensional deformable structure can undermine results especially when loaded [41]. Wirth et al. introduced a 3D method to determine the meniscal position in relation to its contact area and morphology [45]. This method permitted conduct meniscal extrusion globally in all areas of the knee joint. To exhibit motion of the meniscus Yao et al. [46] proposed a 3D matrix volume with calculated centroids. Change of centroids indicated and quantified the meniscal motion.

The 3D voxel vector based approach used in this study permits the evaluation on motion in all parts of the meniscus with direction and range of the movement.

Emerging 3D‐based methods to determine position and motion of the meniscus accurately will improve understanding of meniscal physiological structure and function, aiding the ability to detect the appearance and development of pathologies. Admittedly, the effort put in by manual segmentation has to be decreased by (semi‐) automated segmentation to be a valuable tool in large longitudinal cohort studies.

In vitro studies of the meniscal position and motion rely only on passive stabilisers of the knee joint. Since degenerative changes and different tissue plasticity are present in most cadavers, in vivo studies should be favoured for kinematic analyses [8, 40, 42].

This study examined a relatively young cohort, ensuring intact tissue with no degenerative changes. Although participants were instructed not to resist the movement of the device, active stabilizers were in play at the end of motion. Overall, it depicts physiological in vivo movement of the joint.

There are some limitations regarding this study, especially due to the in vivo setting.

Soft muscle and fat tissue impeded with femoral fixation and allowed the femur to follow movement of the device's footpiece by a short margin.

Despite instruction, evasive movement of the participant to the applied forces could not fully be eliminated.

However, neither of these limitations should significantly affect the relative change in dynamics between tibia and femur because the applied forces first travelled through the joint before being further transferred to the fixation or to body counter parts.

With a cohort of 31 subjects no adapted statistical analyses with co‐dependent factors such as height, BMI or age were performed. Study of a larger longitudinal cohort may provide more differentiated insight.

Thus, we believe the relative improvements in measurement and analysis in this study are sufficient to establish a baseline for assessment of different knee joint pathologies.

## CONCLUSION

As hypothesised, there was movement during application of varus and valgus forces in all compartments of the compressed meniscus towards the outer edge of the tibial cartilage, but the motion was not equally distributed. The AH and PHs contributed most of the motion, thereby showing the plasticity of the meniscal structure. Meniscal motion was found not only in the loaded compartment, but also in the discharged compartment as dynamic intrusion. Analysing the impact of traumatic or degenerative injuries on meniscal motion should be evaluated in future studies.

## AUTHOR CONTRIBUTIONS

Sebastian F. Bendak and Kaywan Izadpanah designed the study and collected data. Andreas Fuchs and Joachim Georgii performed the statistical analysis. Sebastian F. Bendak wrote the manuscript. Sebastian F. Bendak, Jonas Buchholtz, Thomas Lange, Elham Taghizadeh, Stefan Heldmann and Joachim Georgii carried out the measurements and controlled the meniscus segmentation and deformable registration. Kaywan Izadpanah, Andreas Fuchs and Joachim Georgii assisted with statistical analysis and data interpretation, and critically reviewed the manuscript. Hagen Schmal and Moritz Mayr helped with data interpretation and critically reviewed the manuscript. All authors read and approved the final manuscript.

## CONFLICT OF INTEREST STATEMENT

The authors declare no conflicts of interest.

## ETHICS STATEMENT

The study was approved by the institutional review board of the University Hospital Freiburg (Nr. 91/19—210696) and the study was performed in accordance with the Declaration of Helsinki. All volunteers gave written informed consent prior to participation.

## Data Availability

All relevant data are provided within the manuscript. The datasets used and/or analysed during the current study are available from the corresponding author on reasonable request.
